# Serum miRNA as a possible biomarker in the diagnosis of bipolar II disorder

**DOI:** 10.1038/s41598-020-58195-0

**Published:** 2020-01-24

**Authors:** Sheng-Yu Lee, Ru-Band Lu, Liang-Jen Wang, Cheng-Ho Chang, Ti Lu, Tzu-Yun Wang, Kuo-Wang Tsai

**Affiliations:** 1 Department of Psychiatry, Kaohsiung Veterans General Hospital, Kaohsiung, Taiwan; 2 Department of Psychiatry, College of Medicine, National Yang-Ming University, Taipei, Taiwan; 3 Department of Psychiatry, Faculty of Medicine, Kaohsiung Medical University Kaohsiung, Kaohsiung, Taiwan; 4 Department of Psychiatry, College of Medicine and Hospital, National Cheng Kung University, Tainan, Taiwan; 5 Yanjiao Furen Hospital, Hebei, China; 6 Department of Child and Adolescent Psychiatry, Kaohsiung Chang Gung Memorial Hospital and Chang Gung University College of Medicine, Kaohsiung, Taiwan; 7 Department of Medical Education and Research, Kaohsiung Veterans General Hospital, Kaohsiung, Taiwan; 8 Department of Research, Taipei Tzu Chi Hospital, The Buddhist Tzu Chi Medical Foundation, New Taipei, Taiwan

**Keywords:** Diagnostic markers, Bipolar disorder

## Abstract

The diagnosis of Bipolar II disorder (BD-II) is currently based on the patients’ description of symptoms and clinical behavioral observations. This study explored the possibility of miRNA in peripheral blood (serum) as a specific biomarker for BD-II. We identified 6 candidate miRNAs to differentiate BD-II patients from controls using next-generation sequencing. We then examined these candidate miRNAs using real-time PCR in the first cohort (as training group) of 79 BD-II and 95 controls. A diagnostic model was built based on these candidate miRNAs and then tested on an individual testing group (BD-II: n = 20, controls: n = 20). We found that serum expression levels of miR-7-5p, miR-23b-3p, miR-142-3p, miR-221-5p, and miR-370-3p significantly increased in BD-II compared with controls in the first cohort, whereas that of miR-145-5p showed no significant difference. The diagnostic power of the identified miRNAs was further analyzed using receiver-operating characteristic (ROC). Support vector machine (SVM) measurements revealed that a combination of the significant miRNAs reached good diagnostic accuracy (AUC: 0.907). We further examined an independent testing group and the diagnostic power reached fair for BD-II (specificity = 90%, sensitivity = 85%). We constructed miRNA panels using SVM model, which may aid in the diagnosis for BD-II.

## Introduction

Bipolar disorder (BD) has several subtypes. Bipolar I disorder (BD-I) and bipolar II disorder (BD-II) are two of the most commonly seen subtypes of BD. BD-I and BD-II differ in clinical presentation: BD-I involves one or more manic or mixed episodes in addition to one or more major depressive episodes. BD-II is characterised by at least one hypomanic episode with one or more major depressive episodes^1,2^. BD-II has become the focus of increasing attention and has been recognised as a unique disorder. Its prevalence rate ranges from 3% to 11%^3^. Nevertheless, because of the complex spectrum of symptoms, the diagnosis of BD remains subjective. Diagnosing BD is challenging because patients typically regard hypomanic episodes as a positive mood status or experience, and they only seek medical help during depressive episodes^4^. BD is initially misdiagnosed at a rate of approximately 40% and typically requires approximately 5–12 years to be correctly diagnosed and treated appropriately^5,6^. Delayed diagnosis and inefficacious treatment of BD-II may lead to a prolonged course, more affective episodes, and an increased suicide rate, adding further to its socioeconomic burden^5,7^.

Accumulating evidence reveals that microribonucleic acids (miRNAs) are involved in many biological processes such as neurogenesis, neuroproliferation, and synaptic plasticity in the central nervous system^8,9^. Different expressions of peripheral miRNA have been reported in several mental disorders including schizophrenia, major depressive disorder, and Alzheimer disease^10–12^. Changes in peripheral miRNA may be correlated with changes in neuroendocrine or neuroimmune responses^13^. Because brain tissue is not easily accessible, blood-based miRNAs, which are inexpensively, noninvasively, and easily obtained, have been proposed for use as clinically applicable biomarkers of psychiatric disorders.

Because BD may be a neurodegenerative disorder, previously investigated candidate miRNAs mostly involved the regulation of immunity^14^, which may contribute to the pathomechanism of neurodegeneration^15–17^. However, due to methodological inconsistencies, few results have been replicated. In addition, studies on miRNAs have mostly assessed only undifferentiated BD and not BD-II in particular^18^. The role of miRNA in BD-II remains unclear and warrants further study.

The present study explored whether miRNAs are noninvasive and applicable biomarkers that can assist in the diagnosis of BD-II. We identified miRNAs that may be associated with BD-II and evaluated whether they could be proper peripheral biomarkers. Using the identified miRNAs, we plan to develop a diagnostic tool that may yield early accurate diagnosis and effective pharmacological treatment of BD-II.

## Methods

Patients with BD-II and healthy controls aged between 20 and 65 years were recruited from inpatient and outpatient clinics at the Department of Psychiatry, Kaohsiung Veterans General Hospital and National Cheng Kung University Hospital. The research protocol was approved by the institutional review boards of Kaohsiung Veterans General Hospital (VGHKS18-CT9–15) and National Cheng Kung University Hospital (A-ER-107–133). The recruitment procedure followed relevant guidelines. The recruitment procedure was performed following related guidelines. After describing the study purpose to the patients, they agreed and signed the informed consent form.

### Patients and procedures

Patients aged between 20 and 65 years and diagnosed with BD-II by research psychiatrists were recruited after a thorough medical and neurological evaluation. The patients recruited underwent a structural interview to confirm their diagnosis; the Chinese version of the Modified Schedule of Affective Disorders and Schizophrenia-Life Time (SADS-L)^19^ was used. Epidemiologic data revealed that a 2-day duration is more common in community samples^20–25^; therefore, BD-II was diagnosed in the current study using a 2-day minimum criterion for hypomania instead of the 4-day duration adopted in the Diagnostic and Statistical Manual of Mental Disorders, Fourth Edition Text Revision (DSM-IV-TR; American Psychiatric Association, 2000). The inclusion criterion was being diagnosed with BD-II, either for the first time or with previous episodes. Exclusion criteria included (a) any other DSM-IV-TR Axis I diagnosis, such as organic mental disorders, substance use disorder, and other major and minor mental illnesses aside from BD-II, and (b) any significant medical or neurological disorders.

We recruited 118 healthy controls aged between 20 and 65 years from the community. All controls underwent structural interviews using the SADS-L to confirm and screen for psychiatric conditions. The controls did not have major and minor mental illnesses (such as schizophrenia, affective disorder, anxiety disorder, substance use disorder, and personality disorder) and did not have a family history of psychiatric disorder among their first-degree relatives. They had no history of receiving blood transfusions or experiencing severe trauma within the past month.

### Measures of symptomatology

Clinical severity was assessed using the Young Mania Rating Scale^26^ and the Hamilton Depression Rating Scale^27,28^, each of which was administered by research psychiatrists.

### Small RNA library preparation

From the antecubital vein of each participant, 20 mL of whole blood was collected. Few studies have discussed miRNA profiling in BD-II. In this study, we first used samples from three randomly selected BD-II patients and three controls for next-generation sequencing to identify candidate miRNAs for BD-II. Briefly, 350-µL serum samples were prepared following the manufacturer’s protocol and using the NEBNext small RNA library prep kit (Cat#E7300, New England Biolabs, Ipswich, MA, USA). The detailed process for preparing serum samples was described in our previous study^29^. Afterwards, with the Illumina MiSeq platform, the small RNA libraries were sequenced (150 cycle, single read; MiSeq Reagent kit V3_150 cycles; Illumina, San Diego, CA, USA).

### NAnalysis of small RNA sequencing data using bioinformatics

In this study, we explored the small RNA profiles in six libraries (three healthy controls and three patients with BP-II). After sequencing, the clean reads were obtained by removing low-quality reads and 3′ adaptor trimming. Then, clean reads were mapped back to human pre-miRNA (miRbase release 19) using Bowtie 2 tools. Mature miRNAs have many variants with different lengths, known as isomiR. Therefore, a mature miRNA may consist of numerous isomiRNAs. The expression levels of miRNAs were calculated with total read counts of all isomiRs. In addition, a mappable read must range from 18 to 25 nt. in length. Reads with short lengths were removed in our study.

### Real-time quantitative reverse-transcription PCR (qRT–PCR)

Total RNAs were isolated from 250-µL serum of clinical samples and were subjected to quantitative detection of miRNA by using the cDNA TaqMan Advanced miRNA cDNA synthesis kit (Applied Biosystems, Inc., USA). Synthesized cDNA samples were then subjected to qRT–PCR by using the TaqManR Universal PCR Master Mix II and TaqMan Advanced miRNA assays according to manufacturer’s instructions (Applied Biosystems). Expression levels of miRNAs in serum were normalized with miR-16. The following IDs of miRNA were used: hsa-miR-7-5p (483061_mir), hsa-miR-142-3p (477910_mir), hsa-miR-370-3p (478326_mir), hsa-miR-23b-3p (483150_mir), hsa-miR-221-5p (478778_mir), and has-miR-16 (481312_mir).

### Functional annotation of differential expressed miRNA candidates

Differentially expressed miRNA candidates (p < 0.05) were selected from NGS data, and their putative targets were identified by TargetScan. Then, these target genes were subjected to map onto the *Kyoto Encyclopedia of Genes and Genomes* pathways using the Database for Annotation, Visualization, and Integrated Discovery. A hypergeometric test was performed to identify significantly enriched pathways.

### Statistical analysis

Differences in demographic variables and expression levels of miRNA between BD-II patients and controls were analysed using *t* tests for continuous variables and chi-square tests for categorical variables. To assess the specificity and sensitivity of single miRNAs when differentiating BD-II from controls, we analysed receiver operating characteristic (ROC) curves and the area under the ROC curve (AUC). The cut-off values of optimal diagnostic points of the ROC curve were set at the largest Youden’s index (sensitivity and specificity-1). With expression of miRNA from this first cohort as a training set, a diagnostic model was built by support vector machine (SVM) using the selected miRNAs. Replication studies were then conducted on miRNA expression of the testing group to explore the sensitivity and specificity of the diagnostic model. The correlations between level of miRNA and clinical symptoms was calculated with Pearson’s correlation. We used SPSS v22.0 (Chicago, IL, USA) to perform all statistical analyses.

## Results

### Generation of microRNA profiles of BD-II and control groups

**Figure 1:**
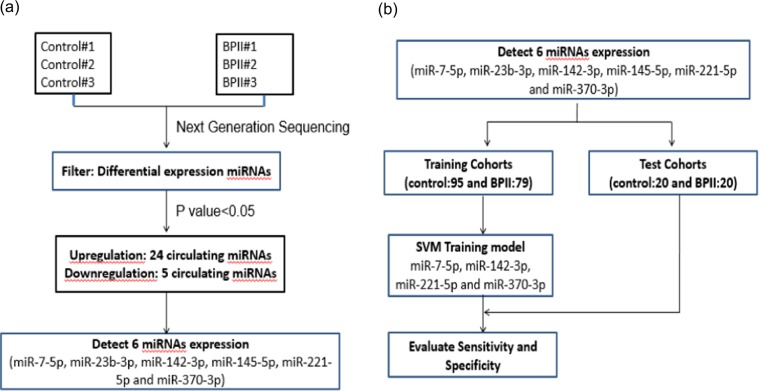
(**a**) Flowchart to identify differentially expressed miRNA candidates through next-generation sequencing (**b**) Flowchart to evaluate the potential of miRNA candidates for diagnostic biomarker.

The study procedure is illustrated in Fig. 1(a,b). We analyzed data of 102 BD-II patients and 118 controls. We randomly selected three patients with BD-II and three controls for the identification of candidate miRNAs. The remaining patients were then divided into training (BD-II, n = 79; control, n = 95) and testing groups (BD-II, n = 20; control, n = 20). The clinical characteristics of all recruited participants are listed in Table 1. All the patients were first diagnosed BD-II and never received treatment for bipolar disorder before. After recruitment and collecting blood samples, the patients were administered medication according to their clinical condition (71.8%, lorazepam; 52.7%, Risperdal; and 89.1%, valproate).

**Table 1:** Clinicodemographic characteristics of patients with bipolar II disorder (BD-II) and healthy controls.

	BD-II	Healthy controls	Χ^2^ or t	P value
n	102	118		
Age (mean, SD)	35.5 ± 11.6	31.0 ± 8.4	3.3	<0.001
Gender (M/F)	38/64	61/57	4.6	0.03
Onset age (years old)	14.5 ± 3.8	N/A		
HAMD	14.2 ± 3.4	N/A		
YMRS	13.1 ± 2.4	N/A		
miR-7-5p (delta Ct)	−17.5 ± 5.3	−22.6 ± 5.8	6.6	<0.001
miR-23b-3p (delta Ct)	−12.3 ± 2.6	−13.3 ± 3.0	2.5	0.013
miR-142-3p (delta Ct)	−3.4 ± 0.8	−5.1 ± 1.4	11.2	<0.001
miR-145-5p (delta Ct)	−3.9 ± 1.4	−4.1 ± 1.7	1.1	0.28
miR-221-5p (delta Ct)	−9.4 ± 0.8	−10.8 ± 1.8	7.6	<0.001
miR-370-3p (delta Ct)	−17.6 ± 8.1	−24.2 ± 6.2	6.6	<0.001

HAMD: Hamilton depression rating scale.

YMRS: Young manic rating scale.

N/A: not available.

SD: standard deviation.

We used t-test for continuous variables and chi-square test for categorical variables.

**Figure 2:**
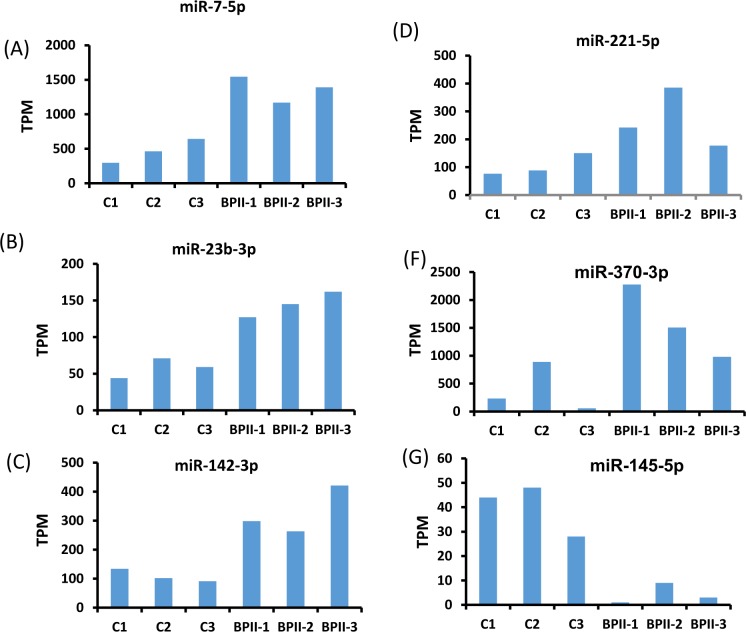
(A–**F**) The expression levels of circulating miRNAs in BD-II samples (n = 3) compared with controls (n = 3). (**A**) miR-7-5p (**B**) miR-23b-3p (**C**) miR-142-3p (**D**) miR-221-5p (**E**) miR-370-3p (**F**) miR-145-5p.

**Figure 3:**
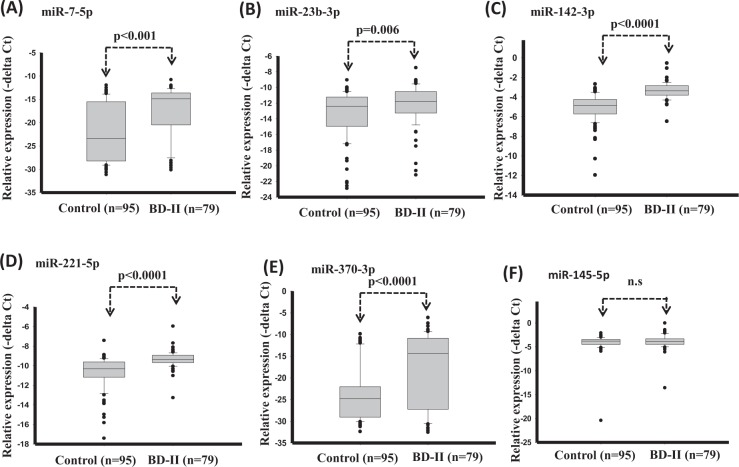
(**A**–**F**) Expression levels of circulating miRNAs in serum in BD-II and healthy controls (Training set). (**A**) miR-7-5p (**B**) miR-23b-3p (**C**) miR-142-3p (**D**) miR-221-5p (**E**) miR-370-3p (**F**) miR-145-5p. (Differences in the expression levels of miRNAs between patients and controls were compared using the t-test).

To identify putative circulating miRNAs as candidates of diagnostic biomarkers for BD-II, we first generated small RNA profiles of three BD-II patients and three controls by using next-generation sequencing (NGS). Both BD-II and control groups comprised two women and one man. The average age was 31.7 ± 5.6 and 38 ± 7.1 years old for the BD-II and control groups, respectively. The three BD-II patients were all drug naïve before entering the study. They were administered mood stabilisers (valproate) and fluoxetine after recruitment and collection of blood samples. We obtained more than 2.2 million clean reads in each sample and identified > 280 circulating miRNAs with > 5 coverage (Supplementary Table 1). The comparison of miRNA expression profiles between control and BD-II samples and filtering steps are as follows: (1) p < 0.05 (2) sums of TPM in BD-II and control ≥100. Five circulating miRNAs were significantly decreased in patients with BD-II compared with controls (p < 0.05), whereas 24 circulating miRNAs were significantly increased in the serum of BD-II patients compared with controls. We selected the five increased circulating miRNAs with the highest significance from these 24 miRNAs: miR-7-5p, miR-23b-3p, miR-142-3p, miR-221-5p, and miR-370-3p. In addition, we selected one significantly reduced circulating miRNA, miR-145-5p, for examination in the additional cohort. The expression levels of these six selected candidate miRNAs in the initial three BD-II patients and controls are displayed in Fig. 2. Because we intended to build a diagnostic model using an SVM, we required a training set to build this model and a testing (validation) set to validate it. Data in the test set were independent from the training set. The samples from the training cohort that included 95 controls and 79 patients with BD-II then underwent real-time PCR. As illustrated in Fig. 3, the expression levels of miR-7-3p, miR-23b-3p, miR-142-3p, miR-221-5p, and miR-370-3p increased more in BD-II patients than in controls, whereas the expression of miR-145-5p exhibited no significant difference.

### Evaluate circulating miRNAs as a diagnostic marker for BD-II

**Figure 4:**
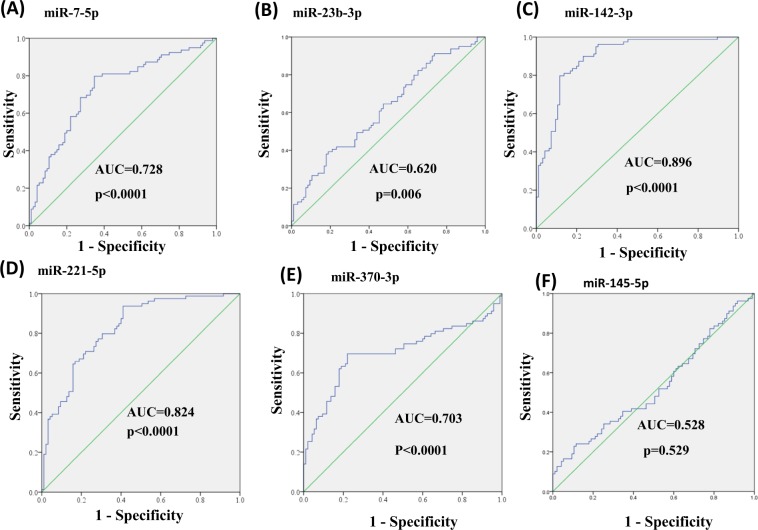
(**A**–**F**) Diagnostic power of the six miRNA candidates using receiver-operating characteristic (ROC) curve analysis. (**A**) miR-7-5p (**B**) miR-23b-3p (**C**) miR-142-3p (**D**) miR-221-5p (**E**) miR-370-3p (**F**) miR-145-5p. (The optimal diagnostic point was assessed at cutoff values with largest Youden’s index (sensitivity and specificity − 1)).

**Figure 5:**
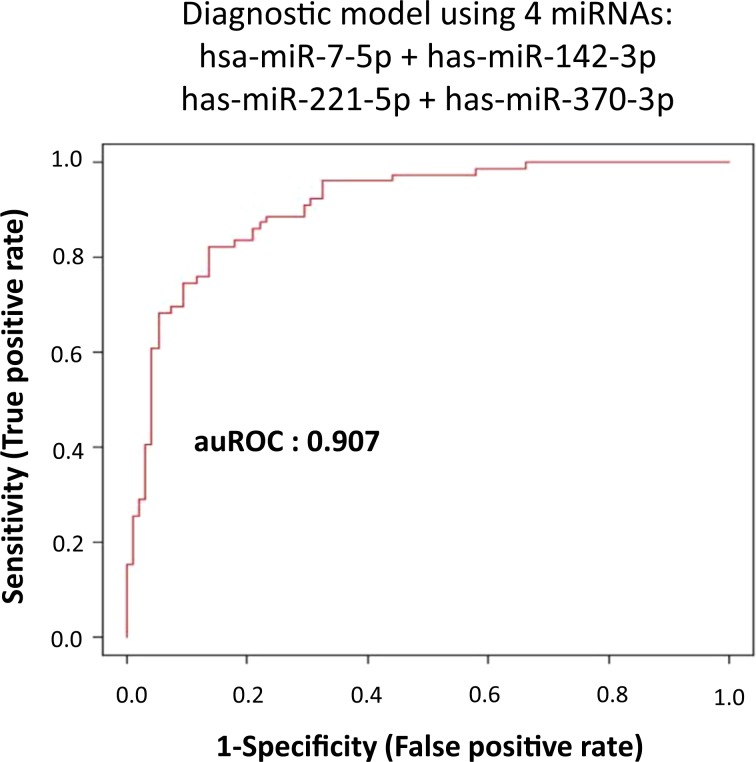
Diagnostic model of BD-II using support vector machine (SVM) (using expression of miRNA miR-7-5p + miR-142-3p + miR-221-5p + miR-370-3p from this first cohort as a training set).

In the previous differential expression analysis, miR-7-5p, miR-23b-3p, miR-142-3p, miR-221-5p, and miR-370-3p expression levels were significantly different between the BD-II and control groups. Therefore, the diagnostic value of using the five circulating miRNAs as potential noninvasive biomarkers for BD-II was assessed and evaluated. We performed ROC curve analysis to determine whether miR-7-5p, miR-23b-3p, miR-142-3p, miR-221-5p, and miR-370-3p could distinguish between BD-II and control groups. As displayed in Fig. 4, the AUC for miR-7-5p was 0.728 (*p* < 0.001), for miR-23b-3p was 0.620 (*p* = 0.006), for miR-142-3p was 0.896 (*p* < 0.001), for miR-221-5p was 0.824 (*p* < 0.001), and for miR-370-3p was 0.703 (*p* < 0.001). The AUC for miR-145-5p remained nonsignificant (*p* = 0.529). By setting a more stringent *p* < 0.05/29 = 0.002 to avoid multiple comparisons, we selected only miR-7-5p, miR-142-3p, miR-221-5p, and miR-370-3p and proposed that these miRNAs may distinguish between individuals with BD-II and healthy controls. We assessed whether the combination of these four miRNA expression data would improve diagnostic power through ROC analysis of probability values using a SVM. We used the expression levels of miR-7-5p, miR-142-3p, miR-221-5p, and miR-370-3p in 79 patients with BD-II and 99 healthy controls as a training cohort to build a diagnostic model using a SVM. The AUC was 0.907 (Fig. 5). Using an independent testing cohort (20 controls and 20 patients with BD-II), we evaluated the accuracy of the proposed miRNA panels. The results revealed a satisfactory outcome in the ability to differentiate patients with BD-II from controls (specificity = 90%, sensitivity = 85%).

We analysed the correlation between level of miRNA and clinical symptoms (Table 2). We noted that miR-23b-3p substantially correlated with the Young Mania Rating Scale score. However, when we corrected for multiple comparison, the correlation became nonsignificant.

**Table 2:** Correlation between level of miRNA and clinical symptoms in all patients with BD-II.

	HAMD		YMRS	
	r	p	r	p
miR-7-5p	0.13	0.21	0.06	0.59
miR-23b-3p	0.14	0.16	−0.28	0.006**
miR-142-3p	−0.12	0.26	−0.08	0.43
miR-145-5p	−0.14	0.18	−0.04	0.67
miR-221-5p	−0.05	0.61	0.05	0.63
miR-370-3p	0.08	0.44	0.05	0.60

HAMD: Hamilton depression rating scale.

YMRS: Young manic rating scale.

r: Pearson’s correlation coefficient.

Correlation between level of miRNA and clinical symptoms was calculated by Pearson’s correlation.

The data supporting the findings of this study are available from the corresponding author upon reasonable request.

## Discussion

Several miRNAs that we identified were able to significantly differentiate patients with BD-II from controls. Our findings on using these candidate miRNAs for BD-II detection are preliminary, and the mechanisms of how these small RNA molecules may affect the pathogenesis of BD-II requires investigation. However, because these miRNAs may play a key role through regulating genes, they have great potential to be useful tools for the diagnosis of BD-II.

We discovered that the upregulation of miR-7-5p may enable discrimination of patients with BD-II from controls. Critically, miR-7 may inhibit the repair of peripheral nerve injury by modulating the migration and proliferation of neural stem cells^30^; additionally, miR-7 was also detected with increased abundance in the neocortex of superior temporal lobes affected by Alzheimer disease^31^. A recent study involving the rat model of herbicide-induced Parkinson disease discovered different expressions of miR-7 in the brain and peripheral blood^32^. The study found upregulation of miR-7 in the brain but downregulation of miR-7 in peripheral blood. The study also revealed that miR-7 regulates the expression of brain-derived neurotrophic factor (BDNF), and its concentration influences the pathophysiology of bipolar disorders through an autoregulatory mechanism. We propose that the link between miR-7 and BD-II may be related to neurodegeneration and BDNF. However, differences in miR-7 expression in central and peripheral systems require further study.

We also found significant upregulation of the serum level of miR-142-3p in patients with BD-II compared with that in controls. Although its relation with BD-II remains unclear, miR-142-3p was first introduced as a hematopoietic-specific miRNA in a mice study. Studies have reported that miR-142-3p may modulate hematopoietic development, including neutrophil development and maturation^33^. In addition, in patients with chronic rhinosinusitis, miR-142-3p may participate in regulation of the body’s inflammatory response (TNF-α expression) through lipopolysaccharide stimulation in human nasal epithelial cells^34^. Because miR-142-3p regulates neuroinflammation through IL-1β-dependent synaptopathy^35^, it may relate to the pathogenesis of other neurodegenerative disorders, such as BD-II. An animal study reported that miR-142-3p may modulate the BMAL1 gene and regulate circadian functions^36^. Although no direct association was found, peripheral (serum) miR-142-3p was revealed to decrease in patients with attention deficit and hyperactivity disorder with a family history of psychiatric diseases^37^. However, its specificity for BD-II requires further study with other mental disorders.

Compared with controls, miR-221-5p was noted to be considerably more upregulated in patients with BD-II. In a recent study, miR-221 was identified to be a potential diagnostic biomarker for atherosclerosis^38^. Although its association with other risk factors for atherosclerosis, such as metabolic syndrome, inflammation, and other mental disorders, remains unclear, our study results supported its association with the pathophysiology of brain diseases.

However, although we discovered that upregulation of miR-370 may discriminate patients with BD-II from controls, no study has reported an association between peripheral miR-370 and mental disorders. Only one previous animal study for depression reported downregulation of miR-370 in brain tissue^39^. The role of miR-370 in BD requires study.

Similarly, although downregulation of plasma miR-23b was reported to be a potential diagnostic and prognostic biomarker in colorectal cancer^40^, the association between miR-23 and mental disorders remains unknown. Recently, miR-23b was noted to be downregulated during encephalomyelitis and may have an anti-inflammatory role in central nervous system inflammation^41^. We noted an inverse correlation between manic symptoms and level of serum miR-23b. Additionally, miR-23b was reported to be downregulated in patients with intracerebral haemorrhages^42^, and an animal study indicated that overexpression of miR-23b in the brain may alleviate neurological functional deficits in rats, possibly due to its anti-inflammatory effects, by modulating neuroinflammation and neuronal protection^43^. Because inflammation may be an etiology for manic symptom, whether miR-23b may be a state marker for manic symptoms merits investigation.

Pathway enrichment analysis revealed that the putative biological function of miRNA candidates that we identified were involved in some neuron degeneration-relative signalling, including GABAergic synapse signalling, glutamatergic synapse signalling, morphine addiction signalling, transformation growth factor (TGF)-beta signalling, Hippo signalling pathways, and FoxO signalling pathways. The identified pathways are related to the nervous system (GABAergic and glutamatergic synapses) and signal transduction (TGF-beta, Hippo, and FoxO signalling), which are all associated with the pathophysiology of affective disorders^44,45^.

The pathway associated with substance dependence (morphine addiction signalling) has never been related to BD; however, because BD is a common comorbid condition with substance use disorder, common genetic underpinnings may be present. Our findings did not replicate the signalling pathways for BD-II identified by Kao *et al*.^46^, probably because of the different approaches implemented. We used candidate miRNAs, whereas Kao *et al*. used single nucleotide polymorphisms identified from genome-wide association scans. Because the pathways associated with BD-II are rarely studied and reported, further study is needed to confirm our results.

The major finding of the current study is the construction of a diagnostic model of BD-II through a combination of miRNAs. The model has a diagnostic power of 0.907 and can differentiate patients with BD-II from controls with satisfactory sensitivity and specificity. Misdiagnosis or delayed diagnosis of BD-II leads to inefficacious treatment, which may increase the risk of rapid cycling and suicide in patients with BD-II^5,7,47^, adding to the burden this disorder places on both patients and society. Biomarkers for BD-II are strongly needed for timely diagnosis and subsequent treatment. Our proposed diagnostic model involving peripheral miRNAs may be an inexpensive, noninvasive, and easily used clinically applicable diagnostic tool for BD-II. However, replication studies using independent study groups or even frequently misdiagnosed mood disorders (such as major depressive disorder and BD-I) are warranted to validate our findings.

Our study has some limitations. First, we sampled serum miRNAs instead of peripheral blood monocytes or central nervous system samples. Our study results should, therefore, be interpreted with caution when applied to other types of samples. Plasma may be the sample of choice when studying circulating miRNA because RNA may be released from blood cells into the serum during coagulation and affect the true status of circulating miRNA^48^. However, miRNAs identified in serum mostly are from exosomes^49^, which are promising biomarkers of psychiatric disorders^50^. Because some samples collected were stored as serum from a previous study^51^, we chose to analyse serum samples. Higher miRNA concentrations may be reported in serum samples than in corresponding plasma samples^48^. Future studies with plasma samples may be necessary to confirm our findings. In addition, although we controlled carefully for other mental disorders, we did not control for common physical comorbidities, such as diabetes, hypertension, and metabolic diseases, which can confound the correlation between miRNA and BD-II. Furthermore, we did not include mood disorders frequently misdiagnosed as BD-II, such as major depressive disorder and BD-I, as comparative groups. Third, our study sample was moderately small, particularly the testing group. Future studies should include a larger sample size and all subtypes of mood disorders. Expression of miRNA may relate to age^52^; however, the temporal window of selected patients and controls was broad. Therefore, our study results should be interpreted with caution. To identify candidate miRNAs that may differentiate patients with BD-II from controls, subjecting the entire sample to NGS analysis would have been ideal. However, our funding was not enough to afford this. Therefore, our results require further replication. In addition, analysing all significant identified miRNAs in the larger cohort would have been ideal. However, to narrow down potential candidates given the time and budget, we only picked approximately the top 20% (the six most likely candidates) for further analysis. With more funding, we could have analysed all the miRNAs identified. Finally, the current cross-sectional design may have precluded evaluation of the role of candidate miRNAs in disease progression and the treatment of BD-II. A longitudinal study may help clarify the pathomechanisms of candidate miRNAs with BD-II and whether they may be used as treatment targets.

## Conclusion

The present study identified miRNAs—miR-7-5p, miR-142-3p, miR-221-5p, and miR-370-3p—that may be associated with BD-II and built a diagnostic model using these potential peripheral biomarkers. We may have developed a novel noninvasive miRNA panel that could act as a possible biomarker for the diagnosis of BD-II. Our proposed diagnostic tool may facilitate early diagnosis of BD-II and timely pharmacological intervention, thus decreasing the burden it places on both patients and society.

## Supplementary information

Supplementary Table 1.
