# Supplementary material for: Serum miRNA as a possible biomarker in the diagnosis of bipolar II disorder
Source: Sci Rep. 2020 Jan 24;10:1131. doi: 10.1038/s41598-020-58195-0 (PMC6981268; doi:10.1038/s41598-020-58195-0)
Supplement: Supplementary file 1 — Supplementary Table 1. [file 41598_2020_58195_MOESM1_ESM.docx]

**Serum miRNA as a possible biomarker in the diagnosis of bipolar II disorder**

*Sheng-Yu Lee, MD, PhD^1,2,3,4,7^, Ru-Band Lu, MD^4,5^, Liang-Jen Wang, MD, MPH, PhD^6^, Cheng-Ho Chang, MD, MS^1^, Ti Lu, MD, MS^1^, Tzu-Yun Wang, MD^4^, Kuo-Wang Tsai, PhD^7,8,^*

^1^Department of Psychiatry, ^7^Department of Medical Education and Research, Kaohsiung Veterans General Hospital, Kaohsiung, Taiwan; ^2^Department of Psychiatry, College of Medicine, National Yang-Ming University, Taipei, Taiwan; ^3^Department of Psychiatry, Faculty of Medicine, Kaohsiung Medical University Kaohsiung, Taiwan; ^4^Department of Psychiatry, College of Medicine and Hospital, National Cheng Kung University, Tainan, Taiwan; ^5^Yanjiao Furen Hospital, Hebei, China; ^6^Department of Child and Adolescent Psychiatry, Kaohsiung Chang Gung Memorial Hospital and Chang Gung University College of Medicine, Kaohsiung, Taiwan; ^8^Department of Research, Taipei Tzu chi Hospital, The Buddhist Tzu chi Medical Foundation, New Taipei, Taiwan

**Supplement Table 1.** Total miRNA reads of initial patients and controls underwent next-generation sequencing

|  | Total reads | Mature miRNA reads | Known miRNA with >5X coverage |
| --- | --- | --- | --- |
| control #1 | 2256226 | 366713 | 374 |
| control #2 | 2521268 | 310705 | 301 |
| control #3 | 2505218 | 300077 | 284 |
| BPII #1 | 2722915 | 287622 | 280 |
| BPII #2 | 2595334 | 549092 | 335 |
| BPII #3 | 4007510 | 588121 | 384 |
